# Physical and technical demands of Australian football: an analysis of maximum ball in play periods

**DOI:** 10.1186/s13102-022-00405-5

**Published:** 2022-01-25

**Authors:** Christopher Wing, Nicolas H. Hart, Fadi Ma’ayah, Kazunori Nosaka

**Affiliations:** 1 School of Medical and Health Sciences, Edith Cowan University, 270 Joondalup Drive, Joondalup, WA 6027 Australia; 2 Institute for Health Research, University of Notre Dame Australia, Fremantle, WA Australia; 3 Caring Futures Institute, College of Nursing and Health Science, Flinders University, Adelaide, SA Australia; 4 School of Education, Curtin University, Bentley, WA Australia; 5 Exercise Medicine Research Institute, Edith Cowan University, Joondalup, WA Australia

**Keywords:** Accelerations, GPS, High-intensity activity, High-speed running, Microsensor technology

## Abstract

**Background:**

This study compares ball in play (BiP) analyses and both whole game (WG) and quarter averaged data for physical and technical demands of sub-elite Australian football (AF) players competing in the West Australian Football League across playing positions.

**Methods:**

Microsensor data were collected from 33 male AF players in one club over 19 games of the 2019 season. BiP time periods and technical performance data (e.g., kicks) were acquired from the Champion Data timeline of statistics, and time matched to the microsensor data. Linear mixed modelling was utilised to establish differences between maximum BiP periods and averaged data.

**Results:**

The analyses indicated significant differences (*p* < 0.0001) between maximum BiP and WG data for all metrics and all playing position (half-line, key position, and midfielders). The percentage difference was greatest for very high-speed running (171–178%), accelerations (136–142%), high-intensity efforts (128–139%), and high-speed running (134–147%) compared to PlayerLoad™ (50–56%) and total running distance (56–59%). No significant (*p* > 0.05) differences were evident for maximum BiP periods when they were compared between playing positions (i.e., half line vs key position vs midfield). Significant (*p* < 0.0001) differences were also noted between maximum BiP phases and averaged data across all 4 quarters, for each microsensor metric, and all playing positions. Technical actions (e.g., kicks and handballs) were observed in 21–48% of maximum BiP phases, depending on playing positions and microsensor metric assessed, with kicks and handballs constituting > 50% of all actions performed.

**Conclusions:**

These results show the BiP analysis method provides a more accurate assessment of the physical demands and technical actions performed by AF players, which are underestimated when using averaged data. The data presented in this study may be used to inform the design and monitoring of representative practice, ensuring that athletes are prepared for both the physical and technical demands of the most demanding passages of play.

## Background

Australian football (AF) is a fast paced, intermittent type sport, characterised by periods of high and low intensity activity [1]. AF players complete large running distances (typically > 12 km) during competitive matches, and perform a vast number of sprints and accelerations as part of these running distances [1–4]. Furthermore, players are also required to perform a number of technical skills (e.g., handballs and tackles) during a match [1]. To understand the physical and technical demands of AF, and facilitate appropriate training prescription, a thorough evaluation of the match running demands needs to be performed.

Match running demands are usually assessed using wearable microsensor technology, inclusive of global position systems (GPS) and micro-electrical mechanical systems (MEMs) within a small unit [1–4] to provide a wide range of accurate and valid data [5]. Oftentimes, these data is presented averaged across quarters, halves or the entire match [2, 3, 6, 7]. For example, the total distance a player has travelled expressed relative to total on-field match time [2, 3, 6, 7], however due to the intermittent nature of AF, this technique underestimates highly intense periods of play [8–10]. Accordingly, there has been a growing need to identify the maximum periods of play, where the physical demands placed upon players are at their highest [9, 10]. Data of this kind can subsequently be utilised to inform the intensity of training prescription that more closely replicates that which is experienced by players during AF matches, thus providing both a physical and technical stimulus that is more likely to transfer to competition [10–12]. This transfer may be aided by improved perception–action coupling, which is more greatly enhanced if training is performed within an environment that closely replicates a match [13–15]. Furthermore, through understanding maximal intensities, coach led training drills can be monitored with greater accuracy [12].

Initially, a fixed time period (e.g., 5 min) was used to perform this assessment; however, more recently a rolling window of time has proven to be a more valid method [8–10, 12]. Specifically, this involves rolling a set time frame (e.g., 5 min) through the raw GPS data at one minute intervals (e.g., minute 1–5, 2–6) iteratively until the end of a match [8–10, 12]. Utilising this method, relative running distances are almost double those derived when averaging data across an entire match, depending upon the analysis window length (e.g., 124 ± 4 vs 226.4 ± 26.4 m·min^−1^) [9].

Despite the added value of the rolling time frame method, the use of non-uniform analysis windows, such as the ball in play (BiP) method, where the analysis period is defined by the natural stop and start of match play, may be able to provide a more detailed description of maximum running periods [11, 16, 17]. The BiP method appears particularly suited to identifying maximum phases in AF, as previous research has demonstrated that the inclusion of data when the ball is out of play reduces relative running performance [18, 19]. Although mean running intensities during BiP in AF have been previously demonstrated [18], the BiP method is yet to be utilised to identify periods of maximum intensity running. In-fact, no method has yet been reported in the literature that assesses maximum running period demands of sub-elite AF players.

Previous research has highlighted match running performance is reduced across match quarters, where typically the highest distances are seen within the 1st quarter, with the lowest recorded during the 4th quarter, likely due to fatigue [2, 20]. Accordingly, it is useful to understand if maximum BiP periods are also similarly affected by fatigue, and whether the magnitude of difference between maximum BiP periods and averaged data remains constant throughout the four quarters. Data of this kind is lacking within the literature.

Additionally, it may be useful to contextualise maximum periods of play with technical match performance (e.g., handballs, kicks). Johnston and colleagues [9] have gone someway to establishing this, reporting maximum relative running intensities across several rolling time frames based upon the number of technical actions performed (kicks, handballs and tackles), such as the maximum intensity of a 1 min period where the player performs 1 action [9]. However, this could be expanded to include other critical actions such as marks, smothers, and spoils, while gaining a greater understanding of how many actions are performed during maximum phases that are derived solely through microsensor data. Having a greater understanding of these technical actions, alongside match running performance, may help coaches design and construct their training prescription to more closely represent AF match demands.

This study aims to compare maximum BiP periods (e.g., the BiP period with the highest meters per minute) and whole game and quarter averaged match running demands of sub-elite AF players across various playing positions. Furthermore, this study aims to provide contextual technical data related to the maximum period of play. It is our hypothesis that these maximum demands would be higher than those derived using match averaged data, and differences will exist between the different positional groups. Furthermore, it is our hypothesis that magnitude of difference between maximum BiP periods and quarter averaged data would decrease from the 1st quarter compared to the 4th quarter, due to fatigue.

## Methods

### Participants

Microsensor data were collected from 33 male sub-elite AF athletes (age: 22.8 ± 3.1 y; mass: 84.2 ± 8.4 kg; height: 184.2 ± 7.6 cm) from one club competing in the 2019 West Australian Football League (WAFL) season over 19 games (15 regular season; 4 finals series), recording 13 wins and 6 losses. Due to the unlimited number of player interchanges permitted in the WAFL, match files (individual player match recordings) were only removed if a player was injured and unable to complete the match or if there was failure of the recording device. A total of 389 match files (average observations per player 12.1 ± 6.6; range 1–19) were included in the final analyses.

Athletes were divided into 7 positional groups, based upon the position they completed the most on-ground time in each individual match. This included full back (n = 3), full forward (n = 5), ruck (n = 3), half back (n = 8), half forward (n = 13), inside midfield (n = 8) and wing (n = 12). Due to issues with sample size using these discrete groups, players were further pooled into 3 general positional groups including key position (full back/forward and ruck, n = 9, match files = 128), half line (half back/half forward, n = 19, match files = 137) and mid-wing (inside midfield/wing, n = 17, match files = 124) in accordance with recent work by others [21]. Due to its potential practical value, descriptive statistics were provided for the 7 discrete groups, however, due to limited sample size, positional statistical comparisons were only made utilising the 3 pooled positions as described above. All participants were provided with the relevant study information before providing informed written consent. The study was approved by the Edith Cowan University Human Research Ethics Committee.

### Procedures

Microsensor data was collected using the PlayerTek device (Catapult Innovations, Melbourne, Australia) sampling at 10 Hz. The accuracy of these devices has been previously confirmed [22]. To reduce interunit variability, players wore the same device throughout the season, fitted within a specifically designed pocket sewed into the playing shirt. All microsensor metrics were expressed per minute of playing time. These included; total running distance (m), high-speed distance (HSR; > 18 km·h^−1^), very high-speed distance (VHSR; > 24 km·h^−1^), PlayerLoad™ (AU), accelerations (efforts > 3 m·s^−2^) and high-intensity efforts (efforts > 18 km·h^−1^ for ≥ 2 s duration). Acceleration efforts were derived from the GPS component of the microsensor device, with a dwell time of 0.5 s. These metrics were selected as they were routinely used in the previous research involving AF populations [3, 9, 10, 23].

Following the completion of each match, data was download onto the proprietary software (Playertek Cloud), with quarter start and end times synced from the PlayerTek live-feed application. Crops were inserted to remove all time periods where a player was on the inter-change bench, as well as periods where a match was stopped for a stretcher (1 occasion during study). This allowed the analysis of on-field time only. This data was subsequently exported to Microsoft Excel (IBM Cooperation, New York, USA) and the data was cleaned and constituted the whole game (from bouncedown to final siren) and quarter averaged data for analysis.

Additionally, Champion Data (Melbourne, VIC, Australia)—a company that provides statistics including coding of events and associated time stamps to both the Australia Football League (AFL) and WAFL—was accessed (with permission) to identify BiP periods and player technical actions. Previous research has found their data to demonstrate acceptable levels of reliability and validity [24]. For the purposes of this study, BiP phases were defined as a period from when an umpire restarts the game with a centre bounce or boundary throw-in or where a player restarts the game with a kick-in, until a time in which an umpire considers the ball to be out of bounds or when the goal umpire signals a goal or behind has been scored. These match events were coded and time-stamped by Champion Data and used to infer BiP periods for this study. Previous research has reported the coding of these events to show acceptable levels of accuracy [25]. In order to accurately time match Champion Data and microsensor data, the lead researcher created a ‘split’ from the bouncedown to the end time of each quarter using live-feed technology (Playertek + live-feed application) in-game, which was automatically synced to the microsensor data. The start time of each quarter was then matched to the bouncedown time stamp (signalling the start of the quarter) provided by Champion Data, which signified the start of the first BiP period for the quarter. Subsequent BiP periods were then manually entered onto the microsensor technology data within the propriety software and exported to Microsoft Excel, for analysis.

BiP data was cleaned by removing all periods of play < 30 s in duration, as periods of this duration appear to give a false indication of intensity [11]. Additionally, all BiP periods where the player did not complete the entire phase of play (i.e., were rotated on or off during the period) were also removed from the final analysis. The maximum BiP period (e.g., the BiP period with the highest meterage or efforts per minute) for each of the 6 metrics outlined above, for every player in every round, and for every quarter, were subsequently used for the final analysis. Player technical actions were manually time matched in Microsoft Excel to the maximum BiP period recorded for each match. These have been described previously [1, 26] and included the following;

- *Kick*: Disposing of the ball with any part of the leg below the knee.
- *Handball*: Disposing of the ball by hand.
- *Tackle*: Using physical contact to prevent an opponent from successfully disposing the ball.
- *Smother*: Suppressing an opposition disposal by affecting the flight of the ball or by blocking the disposal.
- *Spoils*: Knocking the ball away from a contest, preventing the opposition taking a mark.
- *Mark*: Catching a ball that has been kicked when it has travelled > 15 m without being touched by another player or the ground.
- *Hit out*: Tapping the ball out of a ruck contest following a stoppage.

### Statistical analyses

**Fig. 1:**
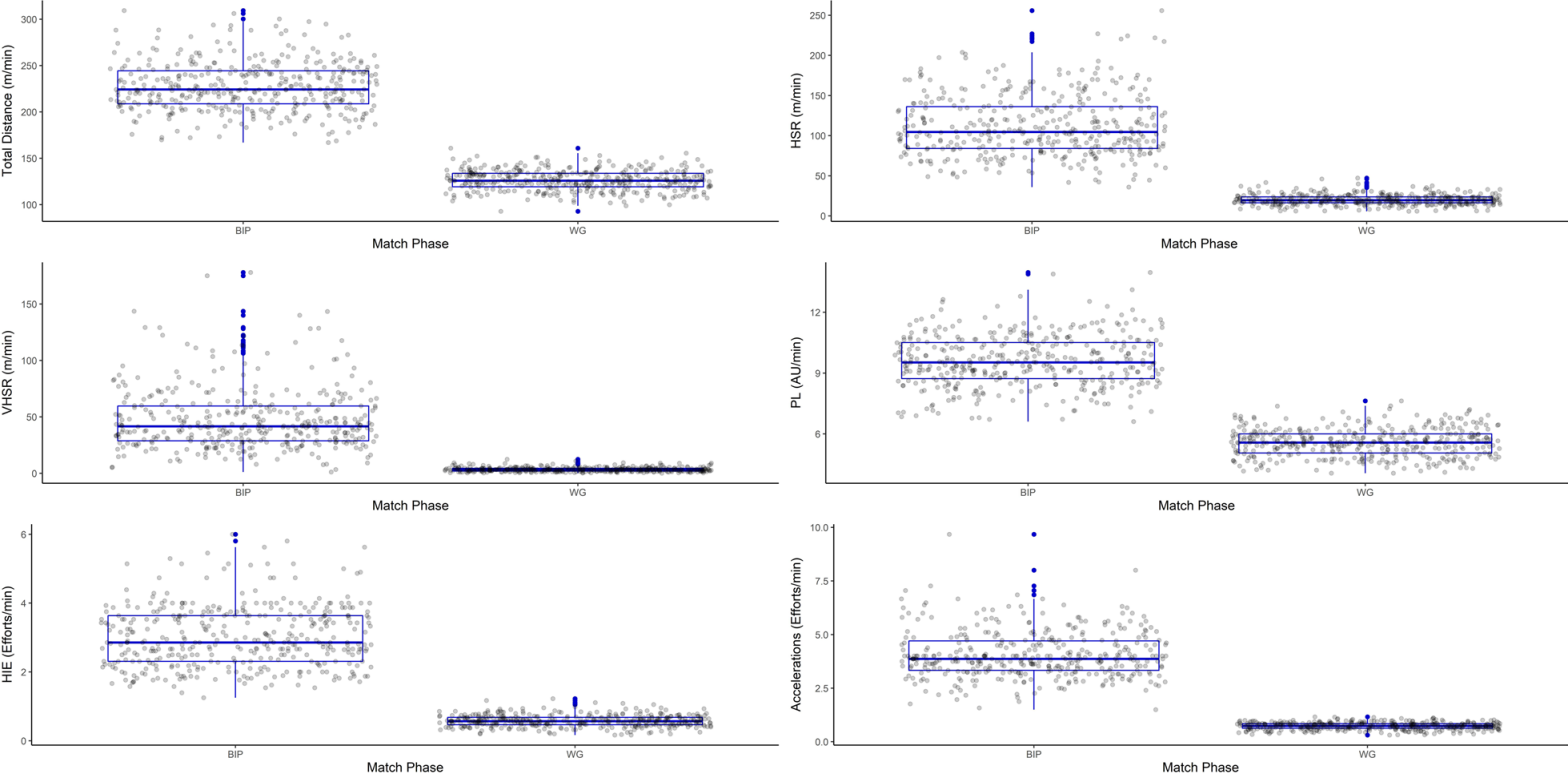
Box plots for comparison of whole game averaged (WG) vs maximum ball in play (BIP) phases for all playing positions combined. Key; HSR: high-speed running (> 18 km·h^−1^), VHSR: very high-speed running (> 24 km·h^−1^), HIE: high-intensity efforts (> 18 km·h^−1^ for ≥ 2 s), PL: PlayerLoad™, Accelerations: (> 3 m·s^−2^)

All statistical analysis was performed in either Microsoft Excel or R software (R, v4.0.4, The R Foundation for Statistical Computing, Vienna, Austria). To assess for differences between maximum BiP and whole game data, as well as between positions, linear mixed models were constructed (lmerTest package), with athlete and round identification included within the model as random effects. The inclusion of athlete identification as a random effect accounts for non-independence of data arising through multiple-observations from the same athlete. Similarly, the same linear mixed model structure was used to assess for differences between maximum BiP periods and data averaged across match quarters. All data was log transformed prior to analysis to reduce error associated with nonuniformity of data [27]. Minor outliers were identified through the construction of box-plots as those 1.5 times outside of the upper or lower interquartile range. However, upon inspection of the raw data points these were shown to be accurate and not errors, and were retained within the final data set [28]. The use of categorical variables for both fixed and random effects ensured that collinearity was not violated. Normality was satisfied through inspection of the QQ plots. However, despite the best efforts of the researchers, and following log-transformation, there was some minor heteroskedasticity remaining within the fitted vs residual plots, which was considered to be weak. Therefore, the confidence intervals reported may be slightly narrower, and should be viewed with an element of caution. This is mainly due to the large differences in the range of data (e.g., BiP has a very large range, whereas averaged data has a very narrow range, see Fig. 1). This does not affect our confidence within the p-values, estimates or ratio’s, and therefore, our conclusion. The visualization of Cooks distance highlighted some influential data points. The models were re-run with these data points excluded, however, there was no change to the significance level of the p-values, therefore the influential data points were retained within the final data set and analyses.

Where significant effects were observed, pairwise comparisons (emmeans package) were utilised with Tukey’s post-hoc test. A separate model was fitted for each measure of running performance and for each match quarter. The significance level was set to *p* < 0.05, and differences were further explained using the pairwise estimates (the adjusted mean difference), ratio’s (the adjusted proportional relationship between averaged data and BiP) and their associated 95% confidence intervals (CI), which were back transformed from the log scale during pairwise comparisons. Additionally, the percentage difference between averaged data and BiP was calculated in Microsoft Excel to the nearest whole percent. The marginal and conditional R^2^ values were also calculated for each model and presented in Table 1.

**Table 1:** Marginal and conditional R^2^ values for the linear mixed models

Outcome variable	Model	Marginal R^2^	Conditional R^2^
Distance (m·min^−1^)	WG v BiP	0.90	0.94
	Q1 v BiP	0.79	0.87
	Q2 v BiP	0.78	0.86
	Q3 v BiP	0.81	0.87
	Q4 v BiP	0.75	0.85
HSR (m·min^−1^)	WG v BiP	0.88	0.93
	Q1 v BiP	0.71	0.82
	Q2 v BiP	0.73	0.82
	Q3 v BiP	0.76	0.84
	Q4 v BiP	0.74	0.82
HIE (efforts·min^−1^)	WG v BiP	0.89	0.94
	Q1 v BiP	0.77	0.85
	Q2 v BiP	0.78	0.84
	Q3 v BiP	0.78	0.84
	Q4 v BiP	0.76	0.83
Player load™ (AU·min^−1^)	WG v BiP	0.82	0.94
	Q1 v BiP	0.69	0.88
	Q2 v BiP	0.69	0.88
	Q3 v BiP	0.73	0.87
	Q4 v BiP	0.65	0.84
VHSR (m·min^−1^)	WG v BiP	0.83	0.91
	Q1 v BiP	0.52	0.68
	Q2 v BiP	0.59	0.72
	Q3 v BiP	0.54	0.67
	Q4 v BiP	0.54	0.66
Accelerations (efforts·min^−1^)	WG v BiP	0.92	0.95
	Q1 v BiP	0.78	0.84
	Q2 v BiP	0.81	0.85
	Q3 v BiP	0.81	0.85
	Q4 v BiP	0.76	0.82

HSR, High-speed running (> 18 km·h^−1^); HIE, High-intensity efforts (> 18 km·h^−1^ for ≥ 2 s); VHSR, Very high-speed running (> 24 km·h^−1^); BiP, Ball in play; WG, Whole game; Q1, 1st Quarter; Q2, 2nd Quarter; Q3; 3rd Quarter; Q4, 4th Quarter

Technical data was presented as number and percentage of maximum BiP episodes where players were required to perform an action. Additionally, all actions were totalled, and a number and percentage were provided for each individual action. These were displayed for each general playing position as well as for each microsensor metric.

## Results

### Whole game vs maximum ball in play

Maximum BiP phases were significantly greater (*p* < 0.0001) for all playing positions across all microsensor metrics when compared with those averaged across an entire game (Tables 2 and 3). The duration of these maximum BiP phases ranged from 30 to 214 s. The difference between maximum BiP periods and whole game averaged data were greater for very-high (ratio: 11.9–17.5, percentage difference: 171–178%) and high-speed running (ratio: 5.0–6.4, percentage difference: 134–147%), and high-intensity (ratio: 4.8–6.0, percentage difference: 128–139%) and acceleration (ratio: 5.0–6.0, percentage difference: 136–142%) efforts, as opposed to total running distances (ratio: 1.8, percentage difference: 56–59%) and PlayerLoad™ (ratio: 1.7–1.8, percentage difference: 50–56%). However, no significant (*p* > 0.05) differences in maximum BiP phases were evident between the playing positions. Figure 1 provides a visualisation of these differences between whole game vs maximum BiP phases where all playing positions have been pooled.

**Table 2:** Mean (± SD) data for whole game and maximum ball in play (BiP) for all playing positions

		Playing positions									
Metric	Match Phase	Full Back (n = 3)	Full Forward (n = 5)	Ruck (n = 3)	Key Position (n = 9)	Half Forward (n = 13)	Half Back (n = 8)	Half Line (n = 19)	Midfield (n = 8)	Wing (n = 12)	Mid-Wing (n = 17)
Distance (m·min^−1^)	Match Average	117.5 ± 8.4	124.1 ± 8.3	114.3 ± 5.9	**119.7 ± 8.8**	129.9 ± 11.0	126.7 ± 10.7	**128.3 ± 10.9**	128.6 ± 10.5	133.3 ± 8.8	**130.4 ± 10.1**
	Max BiP	216.9 ± 24.9	228.5 ± 29.6	199.8 ± 23.5	**219.1 ± 28.2**	235.7 ± 26.1	225.6 ± 24.1	**230.9 ± 25.6**	230 ± 24.3	236.3 ± 23.4	**232.5 ± 24.1**
HSR (m·min^−1^)	Match Average	15.7 ± 3.4	18.5 ± 4.2	8.6 ± 2.2	**15.8 ± 4.9**	23.4 ± 5.0	22.6 ± 4.5	**23.0 ± 4.8**	17.8 ± 3.5	27.5 ± 7.4	**21.6 ± 7.2**
	Max BiP	101.2 ± 34.6	113.6 ± 42.6	76.1 ± 33.0	**102.5 ± 39.6**	119.8 ± 35.6	114.4 ± 34.2	**117.2 ± 34.9**	104.9 ± 33.4	133.4 ± 38.3	**116.1 ± 38.0**
HIE (efforts·min^−1^)	Match Average	0.5 ± 0.1	0.5 ± 0.1	0.2 ± 0.1	**0.5 ± 0.1**	0.7 ± 0.1	0.7 ± 0.1	**0.7 ± 0.1**	0.5 ± 0.1	0.8 ± 0.2	**0.6 ± 0.2**
	Max BiP	2.9 ± 1.0	3.0 ± 1.0	2.0 ± 0.6	**2.8 ± 1.0**	3.1 ± 0.8	3.3 ± 0.8	**3.2 ± 0.8**	2.8 ± 0.7	3.4 ± 0.9	**3.1 ± 0.8**
PlayerLoad (AU·min^−1^)	Match Average	5.3 ± 0.6	5.1 ± 0.4	4.7 ± 0.3	**5.2 ± 0.5**	6.0 ± 0.5	5.4 ± 0.5	**5.7 ± 0.6**	5.7 ± 0.8	6.0 ± 0.6	**5.8 ± 0.8**
	Max BiP	9.6 ± 1.1	9.3 ± 1.0	7.9 ± 0.9	**9.2 ± 1.2**	10.5 ± 1.1	9.1 ± 0.8	**9.8 ± 1.2**	9.3 ± 1.3	10.2 ± 1.3	**9.7 ± 1.4**
VHSR (m·min^−1^)	Match Average	2.3 ± 0.9	3.2 ± 1.3	0.8 ± 0.5	**2.5 ± 1.3**	4.2 ± 1.4	4.0 ± 1.4	**4.1 ± 1.4**	2.0 ± 1.0	5.6 ± 2.4	**3.4 ± 2.4**
	Max BiP	38.0 ± 20.2	55.8 ± 36.1	28.5 ± 21.5	**43.8 ± 29.6**	51.4 ± 26.3	51.7 ± 25.1	**51.6 ± 25.6**	39.0 ± 21.0	60.9 ± 29.8	**47.6 ± 27.0**
Accelerations (> 3 m·s^−2^) (efforts·min^−1^)	Match Average	0.6 ± 0.2	0.7 ± 0.2	0.7 ± 0.1	**0.7 ± 0.2**	0.7 ± 0.1	0.7 ± 0.1	**0.7 ± 0.1**	0.8 ± 0.2	0.8 ± 0.2	**0.8 ± 0.2**
	Max BiP	3.6 ± 1.0	4.1 ± 1.2	4.4 ± 1.4	**3.9 ± 1.2**	4.1 ± 1.2	4.1 ± 1.0	**4.1 ± 1.1**	4.3 ± 0.9	4.0 ± 1.1	**4.2 ± 1.0**

Bold text indicates the general positional groups used for statistical analysis testing

HSR, High-speed running (> 18 km·h^−1^); HIE, High-intensity efforts (> 18 km·h^−1^ for ≥ 2 s); VHSR, very high-speed running (> 24 km·h^−1^)

**Table 3:** Comparison statistics for maximum ball in play (BiP) versus averaged data for whole game (WG), 1st quarter (Q1), 2nd quarter (Q2), 3rd quarter (Q3) and 4th quarter (Q4)

		Playing Position								
		Half-Line			Key			Mid-Wing		
Metric	Period	Estimate (95% CI)	Ratio (95% CI)	Percentage difference	Estimate (95% CI)	Ratio (95% CI)	Percentage difference	Estimate (95% CI)	Ratio (95% CI)	Percentage difference
Distance (m·min^−1^)	WG	101.9 (95.5–108.3)	1.8 (1.7–1.8)	57	99.0 (91.8–106.2)	1.8 (1.8–1.9)	59	101.1 (94.5–107.8)	1.8 (1.7–1.8)	56
	Q1	70.5 (64.2–76.8)	1.5 (1.5–1.6)	41	68.1 (61.5–74.7)	1.5 (1.5–1.6)	43	71.8 (65.2–78.5)	1.5 (1.5–1.6)	42
	Q2	73.7 (67.0–80.4)	1.6 (1.5–1.6)	45	70.0 (62.9–77.2)	1.6 (1.5–1.6)	45	71.1 (64.2–78.1)	1.5 (1.5–1.6)	43
	Q3	82.7 (75.5–89.8)	1.7 (1.6–1.7)	50	77.4 (69.7–85.0)	1.7 (1.6–1.7)	51	82.1 (74.5–89.6)	1.7 (1.6–1.7)	50
	Q4	68.6 (61.9–75.3)	1.6 (1.5–1.6)	44	65.4 (58.2–72.6)	1.6 (1.5–1.6)	44	67.1 (60.1–74.0)	1.5 (1.5–1.6)	43
HSR (m·min^−1^)	WG	89.2 (75.7–102.7)	5.0 (4.6–5.4)	134	82.0 (66.0–98.0)	6.4 (5.8–6.9)	147	93.0 (78.7–107.4)	5.4 (4.9–5.9)	137
	Q1	52.0 (41.7–62.3)	3.0 (2.7–3.3)	102	47.5 (36.0–58.9)	3.8 (3.4–4.3)	119	51.4 (41.1–61.7)	3.2 (2.9–3.6)	106
	Q2	52.5 (42.2–62.8)	3.3 (3.0–3.7)	110	47.7 (36.2–59.1)	4.2 (3.7–4.7)	125	56.6 (45.2–68.0)	3.5 (3.1–4.0)	113
	Q3	60.9 (48.8–73.1)	3.9 (3.5–4.4)	122	54.6 (41.1–68.1)	4.8 (4.2–5.4)	133	62.4 (49.6–75.2)	4.2 (3.7–4.7)	124
	Q4	51.2 (41.3–61.1)	3.5 (3.1–3.9)	113	40.4 (30.9–49.9)	4.1 (3.6–4.6)	122	49.0 (39.2–58.8)	3.6 (3.2–4.1)	116
HIE (efforts·min^−1^)	WG	2.4 (2.1–2.7)	4.8 (4.4–5.1)	128	2.4 (2.0–2.8)	6.0 (5.6–6.5)	139	2.4 (2.1–2.8)	5.0 (4.6–5.4)	135
	Q1	1.4 (1.1–1.6)	2.9 (2.7–3.2)	103	1.6 (1.2–1.9)	4.0 (3.6–4.4)	123	1.5 (1.2–1.7)	3.3 (3.0–3.6)	103
	Q2	1.5 (1.3–1.8)	3.3 (3.0–3.7)	107	1.4 (1.1–1.7)	4.1 (3.7–4.5)	117	1.5 (1.2–1.8)	3.4 (3.1–3.8)	114
	Q3	1.8 (1.5–2.0)	2.6 (2.4–2.8)	123	1.6 (1.4–1.9)	2.7 (2.5–2.9)	126	1.8 (1.5–2.0)	2.6 (2.4–2.8)	120
	Q4	1.4 (1.2–1.7)	3.5 (3.2–3.9)	111	1.3 (1.0–1.6)	4.4 (3.9–4.9)	127	1.4 (1.2–1.7)	3.7 (3.3–4.1)	120
PlayerLoad (AU·min^−1^)	WG	4.0 (3.7–4.3)	1.7 (1.7–1.8)	52	4.2 (3.8–4.6)	1.8 (1.7–1.8)	56	3.8 (3.5–4.1)	1.7 (1.6–1.7)	50
	Q1	3.0 (2.7–3.3)	1.5 (1.4–1.5)	38	2.9 (2.6–3.3)	1.5 (1.5–1.6)	43	2.8 (2.5–3.1)	1.4 (1.4–1.5)	36
	Q2	3.0 (2.7–3.3)	1.5 (1.5–1.6)	42	2.9 (2.6–3.3)	1.5 (1.5–1.6)	44	2.8 (2.5–3.1)	1.5 (1.4–1.5)	38
	Q3	3.3 (3.0–3.7)	1.6 (1.6–1.7)	46	3.4 (3.0–3.8)	1.7 (1.6–1.7)	50	3.2 (2.9–3.6)	1.6 (1.5–1.6)	46
	Q4	2.9 (2.5–3.2)	1.5 (1.5–1.6)	43	3.0 (2.6–3.3)	1.6 (1.5–1.6)	46	2.7 (2.3–3.0)	1.5 (1.4–1.5)	39
VHSR (m·min^−1^)	WG	41.7 (29.3–54.2)	11.9 (10.2–13.9)	171	32.3 (19.6–44.9)	17.5 (14.9–20.6)	178	42.2 (29.5–54.9)	15.1 (12.9–17.8)	173
	Q1	20.1 (12.7–27.5)	4.6 (3.7–5.7)	146	11.6 (6.2–17.0)	5.1 (4.0–6.4)	158	16.4 (10.2–22.7)	4.7 (3.7–6.0)	151
	Q2	21.0 (14.2–27.9)	5.3 (4.3–6.4)	150	11.4 (6.6–16.1)	5.0 (4.0–6.2)	159	18.9 (12.5–25.2)	5.1 (4.1–6.3)	150
	Q3	21.8 (13.9–29.6)	5.9 (4.7–7.4)	159	13.0 (7.1–18.9)	5.1 (4.0–6.5)	161	16.1 (10.0–22.0)	5.4 (4.3–7.0)	161
	Q4	18.5 (12.3–24.7)	5.2 (4.1–6.5)	150	10.1 (5.7–14.5)	4.9 (3.9–6.2)	160	13.3 (8.6–18.1)	4.6 (3.6–5.8)	151
Accelerations (efforts·min^−1^)	WG	3.2 (2.8–3.5)	5.4 (5.1–5.8)	142	3.1 (2.7–3.6)	6.0 (5.6–6.4)	139	3.1 (2.7–3.4)	5.0 (4.7–5.4)	136
	Q1	1.9 (1.6–2.2)	3.6 (3.2–3.9)	114	2.0 (1.6–2.4)	4.0 (3.6–4.4)	122	1.9 (1.6–2.3)	3.3 (3.0–3.7)	110
	Q2	2.1 (1.8–2.4)	3.9 (3.5–4.3)	122	2.0 (1.6–2.3)	4.3 (3.9–4.8)	129	2.0 (1.7–2.3)	3.6 (3.3–4.0)	118
	Q3	2.1 (1.8–2.4)	4.1 (3.7–4.6)	126	2.1 (1.7–2.5)	4.4 (4.0–4.9)	122	2.1 (1.7–2.4)	3.9 (3.5–4.4)	118
	Q4	1.9 (1.6–2.3)	3.9 (3.5–4.4)	122	2.0 (1.5–2.4)	4.4 (3.9–4.9)	127	1.8 (1.4–2.1)	3.6 (3.2–4.0)	114

*P* < 0.0001 for all comparisons made between averaged data and maximum BiP

HSR, High-speed running (> 18 km·h^−1^); HIE, High-intensity efforts (> 18 km·h^−1^ for ≥ 2 s); VHSR, very high-speed running (> 24 km·h^−1^); accelerations (> 3 m·s^−2^)

### Quarters vs maximum ball in play

**Fig. 2:**
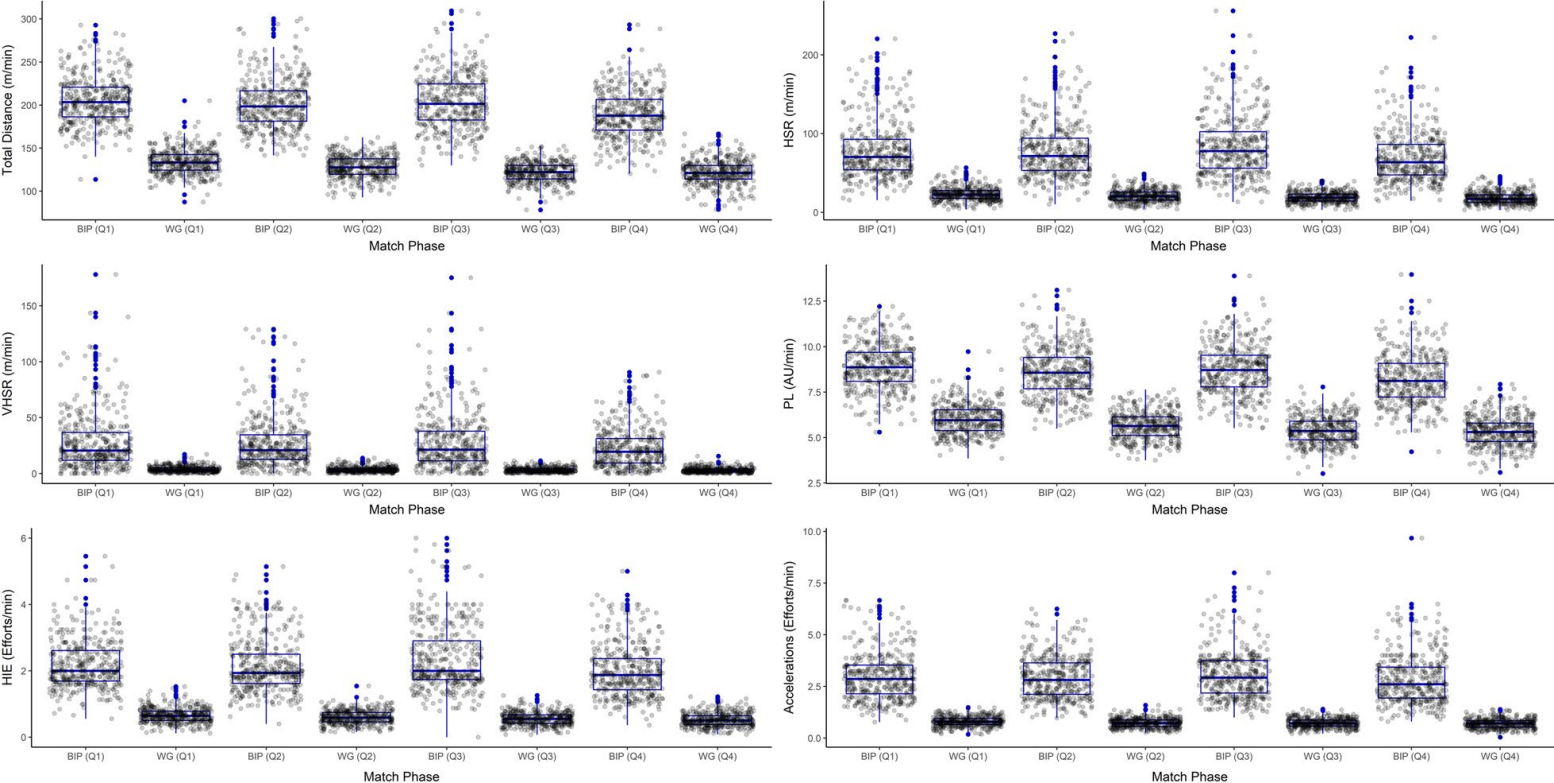
Box plots for comparison of whole game averaged (WG) per quarter vs maximum ball in play (BIP) phases per quarter for all playing positions combined. Key; HSR: high-speed running (> 18 km·h^−1^), VHSR: very high-speed running (> 24 km·h^−1^), HIE: high-intensity efforts (> 18 km·h^−1^ for ≥ 2 s), PL: PlayerLoad™, Accelerations: (> 3 m·s^−2^). WG (Q1): 1st quarter, WG (Q2): 2nd quarter, WG (Q3): 3rd quarter, WG (Q4): 4th quarter

All BiP phases were significantly greater (*p* < 0.0001) for all playing positions, in every quarter, compared to the quarter averaged data (Table 3). The ratio of difference was reasonably similar across the 4 quarters within each playing position; distance (1.5–1.7), high-speed running distance (3.0–4.8), high-intensity efforts (2.7–4.4), PlayerLoad™ (1.5–1.7), very high-speed running distance (4.6–5.9), and acceleration efforts (3.3–4.4), but were lower than those seen when the maximum BiP is compared to the whole game average. Figure 2 provides a visualisation of this data where all playing positions have been pooled.

### Technical actions

Technical actions were performed in 21–48% of the maximum BiP phase from each match, depending on playing position and primary microsensor metric evaluated (Table 4). Generally, more maximum BiP phases containing an action, as well as more total actions performed, were seen during phases defined by the higher-velocity speed banding (e.g., very high-speed running) as well as during those examining maximum acceleration efforts. Additionally, the technical demand was greater among key position and mid-wing players compared to half-line players. Irrespective of the playing position or microsensor technology metric, kicks and handballs constituted > 50% of all technical actions performed. With few exceptions, key position players were required to perform more spoils, marks and hit-outs than mid-wing and half line players across all studied metrics, whereas half-line players were required to perform more tackles. Finally, smothers were rarely seen during maximum BiP phases (0–4%).

**Table 4:** Total number and percentage of maximum ball in play (BiP) periods where an action is performed and number and percentage of each discrete action performed

Maximum BiP Metric	Position (BiP files)	BiP phases where an action is performed	Total actions	Kick	Handball	Tackle	Smother	Spoil	Mark	Hit Out
Distance (m·min^−1^)	Half Line (137)	29 (21%)	39	18 (46%)	7 (18%)	3 (8%)	0 (0%)	5 (13%)	6 (15%)	0 (0%)
	Key Position (128)	40 (31%)	55	23 (42%)	6 (11%)	0 (0%)	0 (0%)	7 (13%)	12 (22%)	7 (13%)
	Mid-wing (124)	34 (27%)	49	23 (47%)	19 (39%)	1 (2%)	0 (0%)	0 (0%)	6 (12%)	0 (0%)
High-speed running (m·min^−1^)	Half Line (137)	33 (24%)	40	16 (40%)	9 (23%)	9 (23%)	0 (0%)	1 (3%)	5 (13%)	0 (0%)
	Key Position (128)	43 (34%)	63	22 (35%)	13 (21%)	1 (2%)	0 (0%)	9 (14%)	10 (16%)	8 (13%)
	Mid-wing (124)	43 (35%)	65	28 (43%)	24 (37%)	4 (6%)	0 (0%)	2 (3%)	7 (11%)	0 (0%)
High-intensity efforts (efforts·min^−1^)	Half Line (137)	37 (27%)	54	19 (35%)	18 (33%)	9 (17%)	0 (0%)	1 (2%)	7 (13%)	0 (0%)
	Key Position (128)	46 (36%)	66	29 (38%)	16 (24%)	3 (5%)	0 (0%)	10 (15%)	6 (9%)	6 (9%)
	Mid-wing (124)	46 (37%)	68	35 (51%)	19 (28%)	5 (7%)	0 (0%)	1 (1%)	8 (12%)	0 (0%)
PlayerLoad™ (AU·min^−1^)	Half Line (137)	37 (27%)	51	24 (47%)	11 (22%)	5 (10%)	0 (0%)	5 (10%)	6 (12%)	0 (0%)
	Key Position (128)	49 (38%)	73	29 (40%)	11 (15%)	4 (5%)	2 (3%)	5 (7%)	12 (16%)	10 (14%)
	Mid-wing (124)	54 (44%)	85	37 (44%)	30 (35%)	6 (7%)	2 (2%)	0 (0%)	10 (12%)	0 (0%)
Very-high speed running (m·min^−1^)	Half Line (137)	35 (26%)	44	22 (50%)	14 (32%)	4 (9%)	0 (0%)	1 (2%)	3 (7%)	0 (0%)
	Key Position (128)	42 (33%)	67	20 (30%)	17 (25%)	2 (3%)	0 (0%)	6 (9%)	13 (19%)	9 (13%)
	Mid-wing (124)	48 (39%)	74	35 (47%)	22 (30%)	8 (11%)	0 (0%)	1 (1%)	8 (11%)	0 (0%)
Accelerations (> 3 m·s^−2^) (efforts·min^−1^)	Half Line (137)	55 (40%)	69	20 (29%)	17 (25%)	18 (26%)	3 (4%)	4 (6%)	7 (10%)	0 (0%)
	Key Position (128)	58 (45%)	77	21 (27%)	11 (14%)	9 (12%)	1 (1%)	9 (12%)	6 (8%)	20 (26%)
	Mid-wing (124)	60 (48%)	85	30 (35%)	26 (31%)	18 (21%)	1 (1%)	2 (2%)	8 (9%)	0 (0%)

HSR, High-speed running (> 18 km·h^−1^); HIE, High-intensity efforts (> 18 km·h^−1^ for ≥ 2 s); VHSR, very high-speed running (> 24 km·h^−1^)

## Discussion

This study examined maximum BiP periods in respect to the physical and technical demands experienced by sub-elite AF players in comparison to whole game and quarter averaged data. As hypothesised, all recorded metrics were significantly greater during maximum BiP phases than those seen across a whole match (Tables 2 and 3). However, in contrast to the hypothesis, no significant differences were noted in maximum BiP phases across the three playing positions.

The values for distance per minute identified within this study were similar to those previously found within maximum periods of play amongst elite level AF players (using the rolling time frame method) [9, 10], indicating that those at the sub-elite level are able to perform similar levels of intermittent high intensity exercise as their elite counterparts. Furthermore, the maximum values for BiP periods were at least comparable, and in some cases greater, to those seen in both professional rugby union [11] and elite youth soccer players [16]. Although all BiP periods were significantly greater than those recorded across a whole game, some metrics displayed a greater increase. For example, total running distance and PlayerLoad™ per minute were approximately 1.6 to 1.8 times higher during BiP periods, whereas very high-speed running was as much as 17 times greater. This may be indicative of the reduced opportunity for athletes to reach and maintain running speeds > 24 km·h^−1^ during a match, owing to reduced pitch spaces afforded by opposition players and therefore increasing the demand to perform several changes of direction and collisions [29], all of which contribute to some form of deceleration and thus reducing maximal running speed. This finding is somewhat corroborated by Wass and colleagues [16] who found relative high-speed (19.8–25.1 km·h^−1^) running performed during BiP periods to show a larger difference than relative distance when compared to averaged data in a population of academy soccer players.

These values reported for maximum BiP periods can be used by practitioners to adequately prepare athletes for periods of high intensity activity [9–12, 16]. In this regard, representative training may be designed and monitored in order to meet the maximum intensities, or a desired percentage of the maximum intensity, recorded during AF matches [30]. For example, a training drill for a key position player at 100% of maximum BiP intensity should be performed at around 219 m·min^−1^, which can be ensured in real time with the use of live-feed GPS technology. This approach ensures that athletes are adequately prepared for the most physically demanding periods of match play, which may not be achieved if training intensities are derived using a whole game approach [30]. Similarly, end-stage rehabilitation drills may also be designed and monitored utilising the same approach, ensuring that athletes are exposed to likely maximal match running intensities before returning to competition, thus increasing their levels of preparedness [16, 30]. Furthermore, it is hypothesised that gaining real-time feedback of the running intensities during BiP periods in competitive matches, through the use of live-feed technology, may have utility in informing interchange-rotation strategies. However, further development and research is required in this space.

Previous research indicates that positional differences exist in physical output during AF matches [3], this was not evident when studying the maximum BiP periods. This suggests that all players are exposed to similar maximum bouts of high-intensity activity of ≥ 30 s in duration. Therefore, it may be beneficial to develop players within a training environment who are adaptable to playing within multiple positions, thus exposing them to a multitude of potential scenarios and problems, which may have a benefit to both player development and the tactical flexibility afforded to AF coaches. Previous research identifying maximal periods of play using rolling time frame methods has shown conflicting evidence regarding positional differences, with Johnston and colleagues [9] finding no effect of playing position, whereas Delaney and colleagues [10] were able to demonstrate differences based upon playing position. Several reasons may be hypothesised for the finding within this study. Most likely, the increased “fluidity” placed upon AF players to play multiple positions within one game, which is particularly evident with the team used in this study. Additionally, differences may have been recognised had the subjects been delineated into smaller positional groups. However, due to sample sizes, this would have required the collection of data across multiple seasons and possibly multiple teams.

When maximum BiP periods and averaged data were compared on a quarter-by-quarter basis, significant differences were still demonstrated (Table 3). This increases the validity of the BiP method when identifying high-intensity periods of play, and thus increases its practical application. Additionally, this finding highlights that athletes are often required to perform periods of high-intensity activity, that are substantially higher than those demonstrated using averaged data, throughout the entirety of a match. Although it had been expected that the magnitude of difference would decrease from the 1st quarter to the 4th, the ratio of difference remained relative stable across the 4 quarters. This may imply that accumulated match fatigue effects a player’s ability to perform all activity, including intermittent bursts of high-intensity activity, to the same degree.

The maximum BiP period from each game were contextualised with technical actions, such as kicks and handballs (Table 4). There was a greater demand on athletes to perform a technical action when the BiP period was defined using higher velocity bands (e.g., very high-speed running). This suggests a player’s ability to produce high velocity outputs may be important to match involvement, and that developing this component of fitness is of importance amongst AF players. This finding could potentially be explained by the work of Sheehan et al. [28], who suggest that links to high velocity movements may be explained by the requirement of players to “beat” their opponents to the ball, or to create space in order to receive the ball from a teammate. This may have important implications for training, where there appears to be a need to create environments where skill execution is performed under match conditions (e.g., speed, execution time, physical pressure) in order to enhance positive transfer [14, 28, 31]. However, it should be noted that previous research has demonstrated that during peak periods of play, average speed was reduced as the number of technical involvements increased [9]. Although this present study demonstrated that more involvements occur in BiP periods defined using higher velocity bands, a cause-and-effect relationship was not established. Therefore, an element of caution should be exercised with this finding.

Additionally, BiP periods defined using acceleration efforts and PlayerLoad™ involved the greatest number of technical involvements, particularly amongst the key position and mid-wing playing groups. As explained by Johnston and colleagues [9], players are often required to perform technical actions within confined spaces, where acceleration load is likely increased, which may go some way to explaining this finding. As PlayerLoad™ is a measure of all accelerations across three movement axis (X = mediolateral; Y = anterior–posterior; Z = vertical) [32], it may be hypothesised that movements such as turning and changing direction are also important to performing a technical involvement. However, further research is required to establish this relationship. Alternatively, the game context may be the greatest factor in the opportunity to perform a technical action. As the majority of BiP periods begin with an umpire re-start (i.e., centre bounce or throw in), players are located within close proximity to the ball, thus increasing their likelihood to perform a technical action.

Some positional differences were noted in respect to technical involvements. With few exceptions, the mid-wing group experienced the greatest technical demand. This is somewhat to be expected when they are often positioned close to the play and their role requires them to “follow” the ball [1, 9, 10]. However, it is maybe surprising that key position players performed more technical actions than the half-line players, especially when they are often confined to smaller areas of the oval [1]. This may be attributable to hit-outs which are only performed by key position players, however, evidence also suggests that they often perform a greater number of marks, kicks and handballs during maximum BiP periods. Due to their position on the field (i.e., near the attacking or defensive goal), these actions may be critical to match outcomes, where they may contribute to a goal being scored or prevented [10]. Additionally, it should also be noted that half-line players may also perform more off the ball actions (e.g., movements that draw defenders to allow greater space for teammates to receive the ball [28]), in order to gain a tactical advantage for the team. Although these do not collect a statistic, these movements are often desirable and may contribute to team success.

These findings regarding technical actions demonstrate the need to integrate both physical and technical development in a combined approach to training. Our findings demonstrate that athletes performed an action in 21% to 48% of maximum BiP phases, suggesting that an action should be included in any representative training drill aimed at replicating these periods of play. As previously mentioned, there is a need to create training environments where athletes are not only exposed to maximal intensities (e.g., meterage per minute), but also to those which require the execution of skill at match pace [14, 28, 31]. This is supported within the current literature which demonstrates that kicking effectiveness is influenced by both time in possession and the level of opposition pressure [31]. Additionally, Ireland et al. [14] demonstrated a disparity in pressure on both the player in possession and the receiver, as well as kick execution time, in current AF training practices compared to competitive matches. Therefore, it is hypothesised that representative training centred around maximal periods of play may go some way to improving current practice design.

## Conclusion

These findings demonstrate that AF players are subjected to periods of high intensity activity across all 4 quarters of a match, which are significantly greater than that seen when the data is averaged. The data presented should be used to inform and monitor the intensity of representative practice and conditioning based drills, enabling practitioners to adequately prepare athletes for the most demanding passages of play [9–12, 16]. As technical actions were performed in 21% to 48% of maximum BiP phases, it is recommended that these are included within training drill prescription that aims to replicate these periods of play. Additionally, the intensities presented within this study may also be used at end stage return to play, ensuring athletes are exposed to likely maximum intensities before returning to performance [16, 30].

## Data Availability

The raw datasets generated during and/or analysed during the current study are not publicly available due to the agreement with the football club.

## Abbreviations

AF: Australian football
WAFL: West Australian Football League
GPS: Global positioning system
BiP: Ball in play
WG: Whole game
HSR: High-speed running
VHSR: Very high-speed running
HIE: High intensity efforts
PL: PlayerLoad^TM^

## References

[1] Johnston RD, Black GM, Harrison PW, Murray NB, Austin DJ (2018). Applied sport science of Australian football: a systematic review. Sport Med.

[2] Coutts AJ, Quinn J, Hocking J, Castagna C, Rampinini E (2010). Match running performance in elite Australian rules football. J Sci Med Sport.

[3] Coutts AJ, Kempton T, Sullivan C, Bilsborough J, Cordy J, Rampinini E (2015). Metabolic power and energetic costs of professional Australian football match-play. J Sci Med Sport.

[4] Johnston RJ, Watsford ML, Austin D, Pine MJ, Spurrs RW (2015). Player acceleration and deceleration profiles in professional Australian football. J Sports Med Phys Fitness.

[5] Scott MTU, Scott TJ, Kelly VG (2016). The validity and reliability of global positioning systems in team sport: a brief review. J Strength Cond Res.

[6] Johnston R, Watsford M, Austin D, Pine M, Spurrs R (2016). Movement profiles, match events, and performance in Australian football. J Strength Cond Res.

[7] Johnston RJ, Watsford ML, Pine MJ, Spurrs RW, Murphy A, Pruyn EC (2012). Movement demands and match performance in professional Australian football. Int J Sports Med.

[8] Varley MC, Elias GP, Aughey RJ (2012). Current match-analysis techniques' underestimation of intense periods of high-velocity running. Int J Sports Physiol Perform.

[9] Johnston RD, Murray NB, Austin DJ, Duthie G (2021). Peak movement and technical demands of professional Australian football competition. J Strength Cond Res.

[10] Delaney JA, Thornton HR, Burgess DJ, Dascombe BJ, Duthie GM (2017). Duration-specific running intensities of Australian football match-play. J Sci Med Sport.

[11] Pollard BT, Turner AN, Eager R, Cunningham DJ, Cook CJ, Hogben P, Kilduff LP (2018). The ball in play demands of international rugby union. J Sci Med Sport.

[12] Whitehead S, Till K, Weaving D, Jones B (2018). The use of microtechnology to quantify the peak match demands of the football codes: a systematic review. Sport Med.

[13] Mann DTY, Williams AM, Ward P, Janelle CM (2007). Perceptual-cognitive expertise in sport: a meta-analysis. J Sport Exerc Psychol.

[14] Ireland D, Dawson B, Peeling P, Lester L, Heasman J, Rogalski B (2019). Do we train how we play? Investigating skill patterns in Australian football. Sci Med Footb.

[15] Tribolet R, Sheehan WB, Novak AR, Watsford ML, Fransen J. How does practice change across the season? A descriptive study of the training structures and practice activities implemented by a professional Australian football team. Int J Sport Sci Coach. 2021; 17(1): 63-72

[16] Wass J, Mernagh D, Pollard B, Stewart P, Fox W, Parmar N, Jones B, Kilduff L, Turner AN (2020). A comparison of match demands using ball-in-play vs whole match data in elite male youth soccer players. Sci Med Footb.

[17] Corbett DM, Sweeting AJ, Robertson S (2019). A change point approach to analysing match activity profiles of team-sport athletes. J Sports Sci.

[18] Rennie MJ, Kelly SJ, Bush S, Spurrs RW, Austin DJ, Watsford ML (2020). Phases of match-play in professional Australian football: distribution of physical and technical performance. J Sports Sci.

[19] Vella A, Clarke AC, Kempton T, Ryan S, Holden J, Coutts AJ (2021). Possession chain factors influence movement demands in elite Australian football match-play. Sci Med Footb.

[20] Aughey RJ (2011). Increased high-intensity activity in elite Australian football finals matches. Int J Sports Physiol Perform.

[21] Thornton HR, Armstrong CR, Gamble T, Rigby A, Johnston RD, Duthie GM. Quantifying the movement characteristics of Australian Football League women’s competition. J Strength Cond Res. 2020. (Published online ahead of print). 10.1519/JSC.0000000000003810 32898037

[22] Mooney T, Malone S, Izri E, Dowling S, Darragh IAJ (2021). The running performance of elite U20 Gaelic football match-play. Sport Sci Health.

[23] Edwards T, Piggott B, Joyce C, Chivers P (2015). The relationship between two measures of physical capacity and match performance in semi-professional Australian rules football. J Aust Strength Cond.

[24] Robertson S, Gupta R, Mcintosh S (2016). A method to assess the influence of individual player performance A method to assess the influence of individual player performance distribution on match outcome in team sports. J Sports Sci.

[25] Rennie MJ, Watsford ML, Spurrs RW, Kelly SJ, Pine MJ (2018). Phases of match-play in professional Australian football: descriptive analysis and reliability assessment. J Sci Med Sport.

[26] Australian Football League. Every stat explained, 2017. Available from: https://www.afl.com.au/news/144837/stats-glossary-every-stat-explained. Accessed Apr 01 2021.

[27] Black GM, Gabbett TJ, Johnston RD, Naughton G, Cole MH, Dawson B (2018). The influence of rotations on match running performance in female Australian football midfiedlers. Int J Sports Physiol Perform.

[28] Sheehan W, Tribolet R, Novak AR, Fransen J, Watsford ML (2021). An assessment of physical and spatiotemporal behaviour during different phases of match play in professional Australian football. J Sports Sci.

[29] Gastin PB, Hunkin SL, Fahrner B, Robertson S (2019). Deceleration, acceleration, and impacts are strong contributors to muscle damage in professional Australian Football. J Strength Cond Res.

[30] Thornton HR, Armstrong CR, Rigby A, Minahan CL, Johnston RD, Duthie GM. Preparing for an Australian Football League Women’s League season. Front Sport Act Living. 2020;23(2). 10.3389/fspor.2020.608939 PMC7785869 33426520

[31] Browne PR, Sweeting AJ, Davids K, Robertson S (2019). Prevalence of interactions and influence of performance constraints on kick outcomes across Australian football tiers: implications for representative practice designs. Hum Mov Sci.

[32] Boyd LJ, Ball K, Aughey RJ (2011). The reliability of minimaxX accelerometers for measuring physical activity in Australian football. Int J Sports Physiol Perform.

